# FOCUS-PDCA can effectively optimize the critical value of test items: FOCUS-PDCA može efikasno da optimizuje kritičnu vrednost ispitivanja

**DOI:** 10.5937/jomb0-34958

**Published:** 2022-07-29

**Authors:** Chunbao Xie, Jianbo Zhang, Jiangrong Luo, Meiling Jian, Taiqiang Zhao, Jiaqiang Wang, Linxi Jiang, Chao Dai, Yao Wei, Li Jiang, Yi Shi

**Affiliations:** 1 University of Electronic Science and Technology of China, Sichuan Provincial People's Hospital, Department of Laboratory Medicine, Chengdu, China; 2 University of Electronic Science and Technology of China, Sichuan Provincial People's Hospital, Department of Operation Management, Chengdu, China; 3 Sichuan Academy of Medical Sciences & Sichuan Provincial People's Hospital, Department of Laboratory Medicine, Chengdu, China; 4 University of Electronic Science and Technology of China, Sichuan Provincial People's Hospital, Sichuan Provincial Key Laboratory for Human Disease Gene Study and Institute of Laboratory Medicine, Chengdu 610072, China

**Keywords:** critical value, optimization, FOCUS-PDCA

## Abstract

**Background:**

To optimize the critical value of test items using FOCUS-PDCA (find, organize, clarify, understand, select, plan, do, check and act), and to set the personalized critical value of the test for different departments.

**Methods:**

We searched for literature reporting on the critical value and FOCUS-PDCA published over recent 5 years in order to understand the significance and status quo of critical value and FOCUS-PDCA. We also collected and analyzed the critical value data of hospital tests performed in Sichuan province hospitals in 2019, which were later compared to data from 2020 to determine the FOCUSPDCA cycle.

**Results:**

The proportion of critical values in the whole hospital decreased from 3.5% before optimization to 2.5% to 3% after optimization. The critical values of ICU, hematology, nephrology, urology, and neonatal departments after optimization significantly decreased compared with those before optimization, while the critical values of cardiac surgery, emergency ICU, cardiology, and neurosurgery ICU showed no significant difference before and after optimization. Contrary, the critical values of the infection department after optimization significantly increased before optimization.

**Conclusions:**

FOCUS-PDCA can effectively optimize the critical value of test items, which is beneficial for rational utilization of medical resources.

## Abstract (sr)

**Uvod:**

Svrha rada je optimizacija kritične vrednosti ispitivanja pomoću FOCUS-PDCA (pronaći, organizovati, razjasniti, razumeti, izabrati, planirati, uraditi, proveriti i delovati), i postaviti personalizovanu kritičnu vrednost testa za različita odeljenja.

**Metode:**

Tražili smo literaturu koja izveštava o kritičnoj vrednosti i FOCUS-PDCA objavljenoj u poslednjih 5 godina da bismo razumeli značaj i status kritične vrednosti i FOCUS-PDCA. Takođe smo prikupili i analizirali podatke kritične vrednosti bolničkih testova obavljenih u bolnicama provincije Sečuan 2019. godine, koji su kasnije upoređeni sa podacima iz 2020. da bismo odredili ciklus FOCUS-PDCA.

**Rezultati:**

Udeo kritičnih vrednosti u celoj bolnici smanjen je sa 3,5% pre optimizacije na 2,5% do 3% nakon optimizacije. Kritične vrednosti odeljenja intenzivne intenzivne nege, hematologije, nefrologije, urologije i neonatalnog odeljenja posle optimizacije su značajno smanjene u odnosu na one pre optimizacije, dok kritične vrednosti kardiohirurgije, urgentne intenzivne nege, kardiologije i neurohirurgije ICU nisu pokazale značajnu razliku pre i posle optimizacije. Naprotiv, kritične vrednosti infektivnog odeljenja nakon optimizacije su značajno porasle pre optimizacije.

**Zaključak:**

FOCUS-PDCA može efikasno optimizovati kritičnu vrednost ispitivanja, što je korisno za racionalno korišćenje medicinskih resursa.

## Introduction

The term critical value was first proposed by Lundberg in 1972 [1], referring to the laboratory test value that is life-threatening to a patient without timely clinical intervention [2]. The item with critical value is called critical value item, while the critical value threshold or critical value boundary is called critical value reporting limit, which refers to the analyte-specific set limits that define a test result as a »critical value [3]. The concept of critical values was endorsed by many countries including China. For example, in 2012 China developed a »medical laboratory quality and criteria for recognition, »which requires that the critical value of clinical laboratory represents a standardized reporting system [4]; yet, at present, no unified critical value of the project and the threshold value has been proposed [5].

Optimizing the critical value reporting process and improving the critical value reporting rate and timely rate of critical value reporting have been explored worldwide. PDCA is a popular iterative methodology that can fix a problem or improve a process and reduce the failure rate of critical value in the laboratory department [6] [7]. The four processes of the PDCA cycle (Plan-Do-Check-Act) are not completed once after running and are carried out repeatedly. Some researchers have applied the PDCA cycle method to test critical value management, effectively reducing the return time of critical value management and medical intervention and improving the critical value registration rate and the qualified rate of registration [8].

FOCUS-PDCA is a novel management mode of continuous quality improvement proposed by American hospital organizations based on the PDCA cycle. It creatively combines FOCUS and continuous cycle improvement (PDCA) and produces a management improvement mode [9] [10] [11] [12]. The characteristics of FOCUS-PDCA are big ring with small ring, step rise, and scientific statistics [13], which are more widely used in patient care, drug management, and medical record management.

This study aimed to optimize the critical value of the test by FOCUS-PDCA and set the personalized critical value of the test for different departments.

## Methods

### Literature research

We searched for literature reporting on the critical value and FOCUS-PDCA published over recent 5 years in order to understand the significance and status quo of critical value and FOCUS-PDCA. Then, we collected and analyzed the critical value data of hospital tests performed in Sichuan province hospitals in 2019, which was later compared to data from 2020to determine the FOCUS-PDCA cycle.

### Find improvement items

The following data were then analyzed: clinical laboratory specimen, critical value of specimen. After the critical value ratio of the whole hospital and all departments in each month was analyzed, the critical value ratio of some departments was too high.

Taking the optimization of critical value as the goal of this improvement project, we expected the project target to be »SMART«, i.e., the target belongs to the specific field of »critical value management«. The proportion of critical value can be used to measure the target situation.

### Organize improvement team

An improvement group, which was set up according to the optimized critical value, included those affected by the excessively high proportion of critical value and those who will be affected by the critical value reform into the group.

### Clarify the current process

According to the current test critical value version, when the LIS system detected the critical value, the test ends are automatically sent to the doctor, and the test staff informs the department and registers the value within the effective time. The critical value items and threshold values for the whole hospital are the same versions and include: blood biochemistry project, blood gas project, coagulation project, and blood routine project.

### Understand analyze the root cause

We hypothesized three fundamental reasons that could lead to a high proportion of critical values: (1) laboratory staff did not know how to optimize critical values on the new system; (2) there were no rules and regulations on the regular optimization of critical values, and there was little communication between clinical departments and clinical laboratory departments on critical values; (3) there was no personalized critical value, and medical staff adopted different clinical treatment methods for patients in different departments.

### Select the improvement plan

We selected the problem points that needed to be improved and set the personalized critical value. The improvement team reviewed relevant literature in the Medical Department and clinical laboratory in early February 2020 and selected the way of »clinical critical value communication meeting« to establish personalized critical value in clinical departments.

### PDCA (Plan-Do-Check-Act)

#### Plan

It was necessary to improve team planning critical value communication to implement specific matters. The project improvement team leader held a critical value clinical communication meeting in the medical department conference room (February 2020) regarding the critical value items and reporting limits. The improvement team members then summarized the critical value communication and informed the medical department (within one week after the meeting). The new critical values were released to the whole hospital after being confirmed by the medical department (March 2020). Soon after that, clinical laboratory and clinical departments held department meetings, respectively, to inform all the staff regarding the new critical value.

#### Do

From February to March 2020, the improvement team successfully implemented the action as planned. In April 2020, the whole hospital began to apply the new critical value. After that, the clinical laboratory regularly communicated the critical value to the clinical department.

#### Check

It was necessary to periodically check whether the LIS system missed the critical value and the understanding degree of laboratory staff to the current critical value version, and to find the following problems: At the beginning of the revision of the critical value version, some staff were not familiar with the new critical value; thus, it was necessary to further adjust the personalized critical value for some departments.

The improvement team collected the critical value specimen information and total specimen information of the whole hospital from May 2020 to March 2021. The proportion of critical value in each month after optimized critical value in the whole hospital and all clinical departments was then counted and compared with the data in 2019. Next, a table was created to observe the difference in the proportion of critical values before and after optimization.

#### Act

After this critical value optimization, the hospital formed a »new version of critical value with personalized critical value«, and the clinical laboratory applied continuous improvement measures of critical value, including the improvement team to evaluate the hospital's critical value periodically every year.

## Results

### Changes of critical value before and after optimization (monthly data)

**Figure 1:**
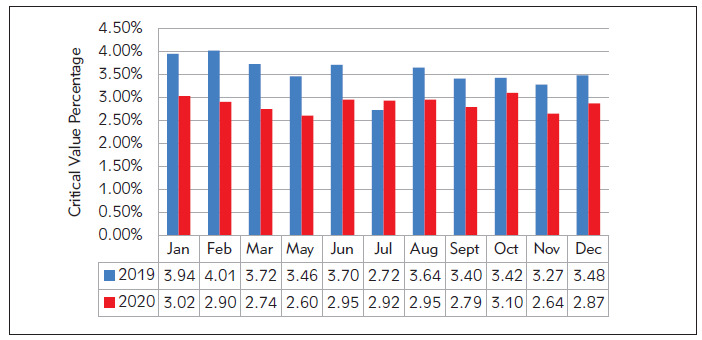
Proportion of critical values of the hospital during 2019 (before optimization) and 2020 (after optimization)

Data from January to March 2019 (before optimization) and 2021 (after optimization) were analyzed and compared. The data from January to March 2020 were actually data from January to March 2021 in order to exclude the impact of the epidemic in 2020. Data from May to December 2019 and 2020 were analyzed and compared. As shown in Figure 1, the proportion of critical values in each month of the hospital decreased from 2.7%–4.1% before optimization to 2.6%–3.1% after optimization.

### Proportion change of critical values in departments before and after optimization

The proportion of critical value in each month before and after the optimization of critical value in each department was counted. The proportion of critical value in some departments decreased significantly, as shown in Table 1 and Table 2. The critical values of ICU, hematology, nephrology, urology, and neonatal departments after optimization decreased significantly compared with those before optimization, while the critical values of cardiac surgery, emergency ICU, cardiology, and neurosurgery ICU showed no significant difference before and after optimization. Contrary, the critical values of the infection department after optimization significantly increased before optimization.

**Table 1:** Change of critical value rate before and after optimization of critical value in each department

Month	ICU		Hematology department		Nephrology department		Organ transplantation center		New pediatric	
	2019	2020	2019	2020	2019	2020	2019	2020	2019	2020
Jan	13.15%	5.54%	15.75%	2.51%	7.65%	3.34%	4.64%	1.77%	7.67%	5.04%
Feb	11.69%	4.89%	16.75%	1.85%	7.89%	3.79%	5.64%	1.73%	8.52%	6.28%
Mar	12.17%	4.53%	19.76%	2.35%	5.93%	3.31%	7.03%	2.21%	7.80%	5.69%
May	12.46%	4.28%	19.65%	2.43%	5.84%	4.08%	6.16%	1.53%	6.91%	7.61%
Jun	12.24%	4.98%	18.94%	3.04%	7.29%	4.12%	4.87%	2.33%	10.98%	8.35%
Jul	11.87%	5.80%	17.50%	3.15%	7.00%	2.93%	2.90%	1.98%	11.83%	8.33%
Aug	13.68%	6.14%	21.19%	2.13%	6.47%	2.98%	7.79%	2.26%	10.23%	6.05%
Sept	12.83%	4.21%	15.74%	2.70%	7.05%	3.96%	5.67%	3.02%	9.85%	6.35%
Oct	11.31%	3.60%	16.96%	3.59%	7.42%	4.08%	4.68%	4.63%	10.59%	6.92%
Nov	11.48%	4.01%	16.66%	2.20%	5.98%	3.65%	6.60%	2.68%	13.29%	8.11%
Dec	13.65%	3.66%	16.71%	2.97%	6.28%	3.41%	7.33%	1.05%	8.89%	7.30%

**Table 2:** Change of critical value rate before and after optimization of critical value in each department

Month	Cardiac surgery		Emergency ICU		Infectious department		Cardiology department		Neurosurgical ICU	
	2019	2020	2019	2020	2019	2020	2019	2020	2019	2020
Jan	2.15%	0.90%	11.27%	10.42%	0.91%	4.99%	5.04%	6.76%	6.20%	4.59%
Feb	1.64%	0.64%	11.02%	10.02%	1.44%	4.33%	5.01%	6.42%	5.16%	3.68%
Mar	2.41%	1.86%	9.31%	11.36%	1.83%	4.46%	4.80%	5.46%	3.89%	4.54%
May	2.44%	2.10%	9.20%	7.25%	0.86%	4.93%	4.70%	4.21%	3.86%	2.84%
Jun	2.60%	1.87%	8.03%	9.76%	1.29%	4.81%	4.89%	5.36%	4.99%	6.02%
Jul	2.05%	1.55%	7.97%	10.46%	1.88%	3.89%	4.61%	5.23%	4.32%	4.43%
Aug	1.92%	2.44%	8.11%	8.71%	1.76%	3.23%	5.31%	5.85%	3.15%	4.96%
Sept	2.31%	2.74%	7.57%	9.22%	1.85%	3.21%	5.22%	6.34%	3.11%	4.58%
Oct	2.61%	2.04%	8.06%	8.32%	1.37%	5.85%	5.25%	6.82%	4.09%	4.10%
Nov	1.54%	1.28%	8.34%	8.52%	1.54%	5.36%	5.06%	5.79%	4.92%	2.70%
Dec	1.63%	1.87%	10.23%	10.00%	1.36%	4.79%	5.23%	6.29%	3.32%	2.14%

**Figure 2:**
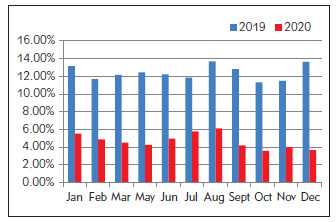
Proportion of critical values of ICU test items before and after optimization

**Figure 3:**
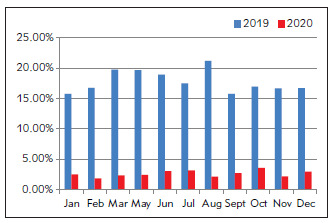
Proportion of critical value before (2019) and after (2020) optimization in Department of Hematology

**Figure 4:**
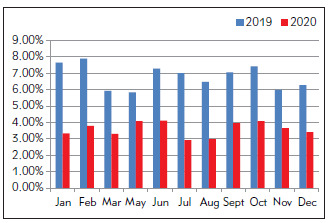
Proportion of critical value before (2019) and after (2020) optimization in Department of Nephrology

The proportion of critical values after ICU optimization was only half of that before optimization. The data from January to March 2020 were actually the data from January to March 2021, as shown in Figure 2. The proportion of critical value after optimization in the Department of Hematology was less than 1/3 of that before optimization (Figure 3), while the proportion of critical value after optimization in the Department of Nephrology was about 1/2 to 2/3 of that before optimization (Figure 4).

**Figure 5:**
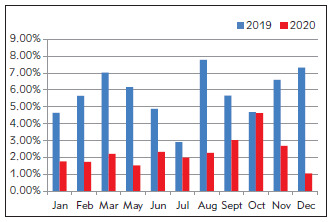
Proportion of critical value before (2019) and after (2020) optimization in Organ Transplantation Center

The proportion of critical value between the adjacent months of external urology in 2019 was significantly different, with the lowest value being 2.90% in July and the highest value being 7.79% in August. However, the optimized data for 2020 and 2021 showed a small difference from month to month that was stable at about 2%. October was an exception, with the critical value ratio reaching 4.63% (Figure 5).

**Figure 6:**
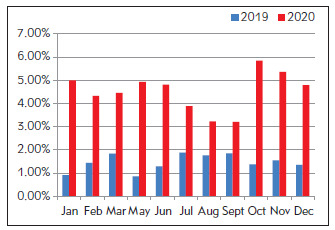
Proportion of critical value before (2019) and after (2020) optimization in Infection Department

Different from the above departments, the proportion of critical values in the Infection Department after optimization could be as low as 1.5 times and as high as 5 times before optimization (Figure 6).

## Discussion

»Critical value« result is an abnormal laboratory test value that is life-threatening to a patient and is reported by laboratory staff based on preset critical limits. It also refers to closely related disease outcomes of test results or to the national major communicable diseases [14] [15].

In China, the concept of critical values has been defined in the Patient Safety Objectives (2014–2015) issued by China Hospital Association in 2014 [16] and Medical Quality Control Indicators for Clinical Laboratory Professionals issued by National Health and Family Planning Commission in 2015. However, standardized optimization of critical values remains a challenge. For example, the effective critical value of auxiliary departments is not consistent with that of clinical identification. Different departments treat the same critical value items differently, and the selection of critical value items and the determination of critical value limits have not been standardized at home and abroad. So far, only a few studies have reported on critical value optimization [17].

AT present, PDCA is the most common method used for critical value optimization. PDCA cycle was first proposed by Hughart, then perfected and popularized by Dr. Deming in 1950 [18] [19]. Many researchers have applied the PDCA cycle to improve management quality [20] [21] [22]. However, PDCA is mainly used to establish critical value reporting procedures or improve the timely rate and qualified rate of critical value in medicine, while it is rarely used to optimize critical value testing projects. Some researchers used the PDCA cycle to improve the registration rate, registration pass rate, and rescue success rate [23], while others used it to analyze the critical value management of hospital clinical laboratory, improve existing problems, and finally improve the standardization of critical value management of hospital clinical laboratory [24]. After applying PDCA to manage critical value, laboratory staff's working attitude, test quality, safety awareness, and operation standardization score have been improved [25].

FOCUS-PDCA is relatively a new approach developed by Hospital Corporation of America (HCA) and used to improve processes. It is mainly used in drug management, patient care, and medical record management, while it is rarely applied for critical value optimization management. Some researchers applied FOCUS-PDCA to solve drug management problems after investigating the procurement, allocation, and use of essential national drugs in the Affiliated Hospital of Nantong University [26]. Also, the application of FOCUS-PDCA significantly reduced the dispensing error rate in pharmacies [27]. Moreover, some studies applied the FOCUS-PDCA cycle method to effectively reduce the incidence of drug proximity error [28].

In terms of patient care, FOCUS-PDCA has been used to effectively solve the difficulties in moisture management of maintenance hemodialysis patients during dialysis [29]. Some researchers also applied FOCUS-PDCA to treat trauma patients, effectively improving the treatment rate of patients [30].

Furthermore, FOCUS-PDCA can reduce the incidence of unreasonable medical orders of parenteral nutrition [31]. In addition, the application of this model effectively improves the completion rate of the first page of inpatient medical records [32]. This circulation can also improve the overall management of blood [33] and help medical institutions achieve significant process improvement [10].

Other researchers adopted FOCUS-PDCA during the epidemic to improve the capacity of hospitals dealing with COVID-19 outbreaks [34] [35] [36]. Previous studies have also suggested that FOCUS-PDCA effectively improves the conversion rate of intravenous drug preparations and reduces medical costs [10].

FOCUS-PDCA has been rarely applied for critical value optimization management. The aim of this study was to optimize the critical value of test items using FOCUS-PDCA (find, organize, clarify, understand, select, plan, do, check and act) and to set the personalized critical value of the test for different departments. Our improvement team collected the total number of clinical specimens and critical value specimen information of each department in Sichuan Provincial People's Hospital in January 2020 and analyzed the change of critical value proportion of each department in the whole year and each month in 2019. The new version of the critical value was implemented in April 2020. The critical value and total specimen data from May 2020 to March 2021 were collected in April 2021 and then compared to data from 2019. In 2020–2021, the hospital's critical values (each month) were 2.5%–3%, while in 2019, the proportion was 3.5%, indicating a significant decrease in the value.

In some representative departments, such as ICU, hematology, nephrology, external urology, and neonatology, critical values decreased significantly after optimization. A particular decrease has been observed in the ICU and the department of hematology (ICU: 12% before optimization to about 4% after optimization; department of hematology from 15% ~ 22% to 1.5% ~ 3.6%).

The optimization of critical value items and their threshold values is not to reduce the proportion of critical value, but to set the critical value according to specimen items, item values, and department personalization. For example, no significant change in critical values was found in the cardiac surgery and emergency ICU department before and after optimization. Interestingly, the critical value in the infection department increased after optimization (less than 2% before and 3%-6% after optimization). Because the infection department has narrowed the range of critical coagulation values.

To sum up, a new version of critical value with personalized critical value has been formed in the hospital using FOCUS-PDCA. The clinical laboratory has effectively optimized the critical value items and their boundary values, screened out the critical value that can truly reflect the critical state of patients in each clinical department, and established continuous improvement measures for the critical value.

## Dodatak

### Conflict of interest statement

All the authors declare that they have no conflict of interest in this work.

### Research funding

This work was supported by the NationalNatural Science Foundation of China (81870683, 82121003), the Department of Science and Technology of Sichuan Province (2020JDTD0028), the CAMS Innovation Fund for Medical Sciences (2019-12M-5-032).
